# Solid State Electronic Sensors for Detection of Carbon Dioxide

**DOI:** 10.3390/s19183848

**Published:** 2019-09-06

**Authors:** Ami Hannon, Jing Li

**Affiliations:** 1 KBR Wyle Inc. at NASA Ames Research Center, CA 94035, USA; 2 NASA Ames Research Center, CA 94035, USA

**Keywords:** carbon dioxide sensor, CO_2_ sensor, gas sensor, room temperature gas sensing, functionalized nanotubes, electronic nose, smartphone sensor, chemiresistive sensor, nanocomposite

## Abstract

Detection of carbon dioxide (CO_2_) is very important for environmental, health, safety and space applications. We have studied novel multiwall carbon nanotubes (MWCNTs) and an iron oxide (Fe_2_O_3_) nanocomposite based chemiresistive sensor for detection of CO_2_ at room temperature. The sensor has been miniaturized to a chip size (1 cm × 2 cm). Good sensing performance was observed with a wide detection range of CO_2_ concentrations (100–6000 ppm). Structural properties of the sensing materials were characterized using Field-Emission Scanning Electron Microscopy, Fourier-Transform Infrared and Raman spectroscopies. The greatly improved sensitivity of the composite materials to CO_2_ can be attributed to the formation of a depletion layer at the p-n junction in an MWCNT/iron oxide heterostructure, and new CO_2_ gas molecules adhere to the high surface area of MWCNTs due to the concentration gradient. The test results showed that the CO_2_ sensor possesses fast response, compact size, ultra-low power consumption, high sensitivity and wide dynamic detection range.

## 1. Introduction

There is a great demand for an effective solid-state electronic device to monitor carbon dioxide (CO_2_) in a variety of applications such as global warming, air quality control, healthcare, mining and the food industry. CO_2_ detection is crucial at the international space station in the crew cabin for the astronaut’s health and safety. The CO_2_ sensor can be used to monitor the CO_2_ concentration in the air of the crew cabin during CO_2_ sequestration processes to make sure that CO_2_ is scrubbed. CO_2_ is a harmful pollutant at higher concentrations due to its ability to displace oxygen in large concentrations. There is about 0.04% (400 ppm) CO_2_ present in ambient air and it is harmless, but once concentration surpasses 1%, it begins to have harmful effects on the human body [1,2]. Headaches start within a few hours of CO_2_ levels of 2–3%. At 4–5% CO_2,_ dizziness, increased blood pressure and breathing issues take hold. Levels above 5% begin to incapacitate the worker. Coma and possible death can occur within minutes at 17% CO_2_. The Occupational Safety and Health Administration’s permissible exposure limit for CO_2_ is 0.5% (5000 ppm) averaged over an 8 h work day [3]. CO_2_ toxicity can be extremely dangerous for workers in confined environments, such as the crew cabin in space, an underground mine and the crew cabin in a submarine. The dangers of this gas when inhaled prompt the need for a sensor that can identify and alert immediately and accurately as soon as CO_2_ concentration reaches toxic levels.

The state-of-the-art commercial sensors for CO_2_ are nondispersive infrared (NDIR) sensors [4]. However, they have issues with precision at different temperatures, pressures and high humidity levels. The NDIR sensor works by comparing how much IR light is absorbed by CO_2_ with how much was emitted to correlate its ratio to CO_2_ concentration_._ The accuracy becomes problematic when the gas absorption lines begin to broaden due to local effects of humidity. At higher pressures, temperatures and humidity levels, radiating and collision time increase, causing the absorption lines to be broaden [5]. Although the NDIR sensor has been miniaturized lately, it is still much larger compared with the electronic sensor that can be made in chip size. In nanotechnology research, sensors for CO_2_ are currently being developed such as silicon nanowires, Sn_2_O_3_ microspheres and polymer nanofilms [6,7,8]. Metal oxides have been used extensively in commercial sensors for many years. They provide high sensitivity to different gases and offer quick response time. Their issue is that they need high operating temperatures, which can be dangerous in flammable environments and also draw large amounts of power [9]. Polymer sensors suffered low sensitivity due to their swelling mechanism for gas sensing. This gives evidence for the need for a more reliable and versatile sensor with a quick response time, wide detection range, in situ monitoring, low power consumption, operation at room temperature and small size.

Carbon nanotubes (CNTs) are an extremely useful sensing material due to their mechanical and thermal stability, electrical conductivity and the ability to adsorb gases [10]. It is a one-dimensional material, which allows almost its entire surface to be available for gas adsorption and charge transfer process [11]. Therefore, it becomes possible to achieve high sensitivity, down to <1 ppb, and it can react to a single foreign gas molecule [12,13]. The electrical characteristics of CNTs are heavily influenced by gases that donate or accept electrons [11]. CO_2_ is a weak oxidizing gas and once it interacts with the CNTs, it will take electrons from the material [14], which is reflected in a measurable resistance change of the carbon nanotubes. Carbon nanotubes have a very high surface area to volume ratio [13], which can be used to improve the sensitivity by using less mass. Pristine CNTs do not contain functional groups, which are needed for specific gas adsorption. It is possible to functionalize and/or dope the CNTs, or to create CNT composite materials that can adsorb a particular type of gas molecules to improve the selectivity [13].

We have developed a composite material using multiwall-carbon nanotubes (MWCNTs) and iron oxide for CO_2_ detection. The carbon nanotubes can adsorb more CO_2_ molecules due to their large surface area, and the iron oxide nanoparticles act as the binding sites to interact with CO_2_ and cause the resistance change due to the charge transfer between the MWCNT/iron oxide and CO_2_. Our MWCNT/iron oxide composite allows operation at room temperature and maintains a sensitive and fast response to CO_2_ [12,13] with a small footprint. Our sensor has its uniqueness, such as small size of 1 cm × 2 cm, low power consumption of micro watts and 2-terminal current-voltage measurement that can be easily multiplexed and integrated with existing electronics. This device can be used with wired and wireless network sensing.

This paper describes a chemiresistive sensor utilizing the selective properties of a MWCNT/iron oxide composite to detect CO_2_ gas from 100 ppm to 0.5% CO_2_ (OSHA exposure limit).

## 2. Materials and Methods

Sensing Materials Synthesis. Multi-wall carbon nanotubes (MWCNTs) were purchased from Nanostructured & Amorphous Materials, Inc. (Houston, TX, USA) and iron oxide was purchased from US Research Nanomaterials, Inc (Houston, TX, USA). All other chemicals such as nitric acid (HNO_3_) and sulfuric acid were obtained from Sigma Aldrich. MWCNTs were first oxidized using mixed acid. MWCNTs were refluxed in mixed acid consisting of concentrated sulfuric acid (98% H_2_SO_4_) and nitric acid (68% HNO_3_) with the volume ratio of 3:1, at 120 °C for 2 h. Dilution, decantation and centrifugation were repeated, followed by rinsing it in water. The purified MWCNTs were then dried at 125 °C for 3 h using a programmable oven. Well dispersed solutions of the oxidized MWCNTs were made in water. Composite materials were prepared by varying weight percentages of the iron oxide (Fe_2_O_3_) in the MWCNTs. Four different composite materials were prepared based on iron oxide weight percent—5%, 4%, 3% and 2%.

Sensor Chip Preparation. The substrate of the sensor chip (1 cm × 2 cm) is made by a grade FR-4 printed circuit board (PCB). This sensor chip consisted of 16 pairs of gold interdigitated electrodes (IDE) screen printed on the PCB substrate. Each pair of IDE is called a sensor or a channel that has the following dimensions: finger width of 70 µm, finger gap sizes of 100 µm.

The sensing material of the oxidized MWCNT/iron oxide composite was deposited onto the chips by drop casting using a micropipette. Each IDE array had 0.3 µL of the composite materials in aqueous solution. A total of 8 channels were deposited with two different sensing materials. Four sensors (channels 1–4) were coated with the oxidized MWCNT/iron oxide nanocomposite and the next four (channels 5–8) were coated with oxidized MWCNTs without iron oxide for comparison. For the sensing material selection, all 16 channels of another chip were used by depositing 4 different composites of MWCNT/iron oxide at different ratios.

**Figure 1:**
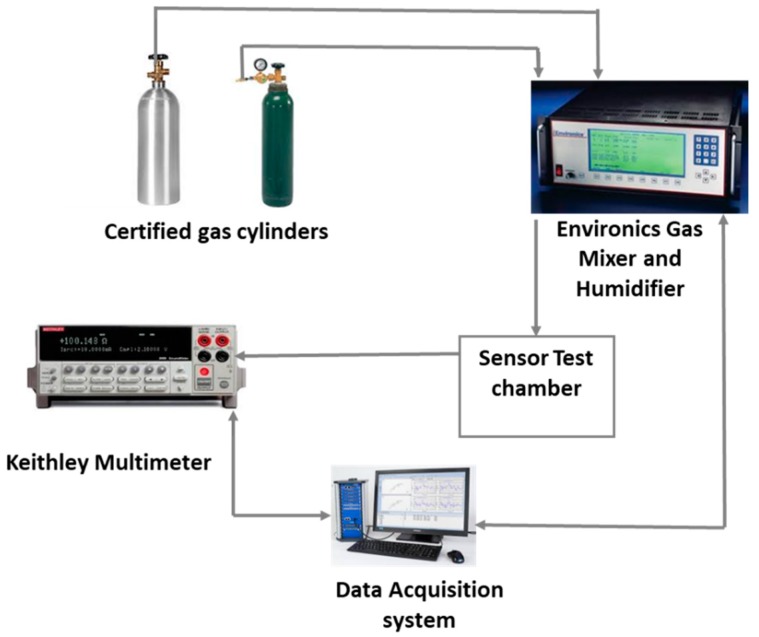
Gas sensor test set-up.

Sensor Testing. CO_2_ gas exposure tests were conducted by attaching a sensor chip to an adapt board and then to a Keithley 2700 (Keithley Instruments, Inc., Scottsdale, AZ, USA) to measure the electrical resistance value of each channel on the sensor chip. An Environics 2000 (Environics Inc., Tolland, CT, USA) gas blending and dilution system is used for introducing CO_2_ gas at different concentrations in air. The certified gases supplied were 10,000 ppm CO_2_ (Matheson) and Zero Air (Praxair). A Teflon cover with a nozzle combined with the sensor chip adapt board was used to form a test chamber for introducing the CO_2_ gas stream directly onto the surface of the sensor chip. The gas stream of 400 cm^3^/min was used for CO_2_ gas exposure and sensor testing. The sensor testing setup is depicted in Figure 1. During the experiment, an initial trial of dry air flew onto the sensor chip for 10 min to establish a baseline for the following CO_2_ exposure and dry air flush cycles. The gas stream of CO_2_ in air was introduced to the sensor chip for 1 min followed by a 5 min dry air flush. The CO_2_ concentrations were increased from 100 ppm to 6000 ppm, and each concentration of the CO_2_ exposure was for 1 min followed by a 5 min dry air flush. A repeatability test was conducted following the same schedule except instead of increasing concentration of CO_2_; it stayed at 4000 ppm consistently. A test of step response with increasing CO_2_ concentration and an air purging at the end of the test was also conducted.

## 3. Results and Discussion

### 3.1. Optimization of Sensing Material

**Figure 2:**
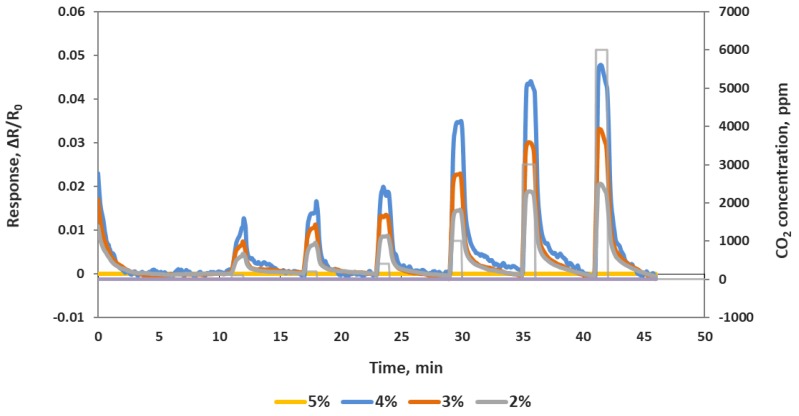
Response of a sensor chip to 100, 200, 400, 1000, 3000 and 6000 ppm CO_2_. Each line is an average of full identical channels.

First of all, to optimize the ratio of the composite sensing material, four different composite sensing materials with oxidized MWCNTs were prepared by varying the iron oxide weight percentage. A sensor chip containing 16 IDEs was used for the deposition of these four different materials. Each material was deposited on four channels by drop casting. After room temperature drying and vacuum oven drying to remove any remaining water, the resistances of all the channels were measured. Channels 1–4, which were deposited with 5% (by weight) iron oxide, did not show any conductance (an open circuit) due to the amount of non-conductive iron oxide nanoparticles in the composite. Channels 5–8 were deposited with 4% iron oxide and displayed an average resistance of ~123 KΩ. Channels 9–12 deposited with 3% iron oxide showed an average resistance of ~3.8 KΩ. Channels 13–16 deposited with 2% iron oxide showed an average resistance of ~0.7 KΩ. This chip was then exposed to various concentration of CO_2_ in the ranges of 100–6000 ppm at room temperature, as shown in the Figure 2. All three composite materials (4%, 3% and 2%) responded well to CO_2_, but the sensitivity to CO_2_ decreased as the iron oxide percent decreased. Sensor channels with 4% iron oxide showed highest relative change to CO_2_ exposure compared to the other three materials, but the higher base resistance made it noisier compared to other compositions. Based on this data, we decided to go with 3.5% iron oxide for further tests as that showed a reasonably high sensitivity and a stable base resistance.

For the rest of the CO_2_ study, we used the 3.5% oxidized MWCNT/iron oxide composite material. We prepared a sensor chip with four channels of the composite material and four channels of oxidized MWCNTs without iron oxide.

### 3.2. Characterization of Sensor Elements

**Figure 3:**
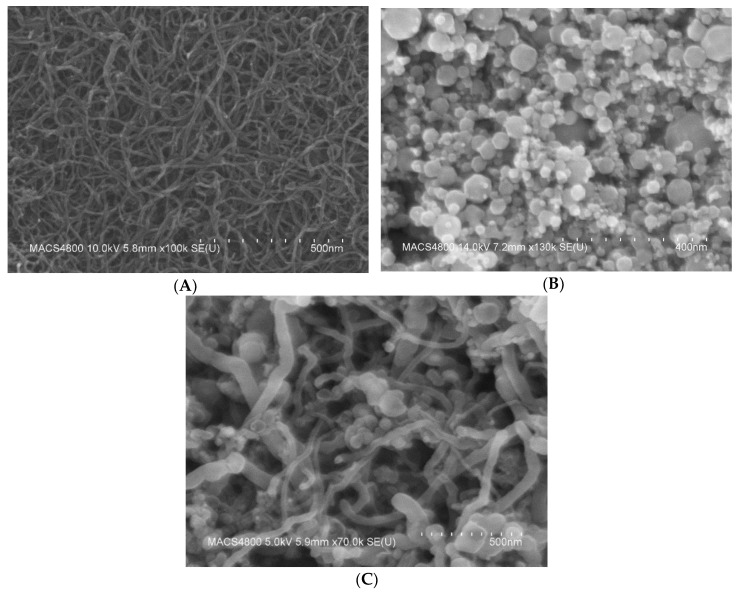
FE-SEM images for (**A**) oxidized MWCNTs deposited onto a silicon substrate, (**B**) iron oxide nanoparticles and (**C**) oxidized MWCNT/iron oxide composite material.

The morphology of the oxidized MWCNT, iron oxide nanoparticles, and the nanocomposites of oxidized MWCNT/iron oxide were investigated using a FESEM Hitachi S-4800 SEM. As shown in Figure 3, it can be seen that the MWCNTs form bundles due to the strong Van der Waals force. MWCNT diameters ranged from 4–10 nm. The image of the iron oxide presents clusters of the iron oxide nanoparticles that cling together and form aggregates in spherical shapes with individual sizes ranging from 5 to 50 nm. The clusters of the iron oxide result from the magneto static coupling between particles [15]. The SEM images of the oxidized MWCNT/iron oxide nanocomposites show networks of MWCNT interwoven among the iron oxide nanoparticles. A research group has reported that the composite material is not just a mechanical mixture of the components, but they have detected chemical bonds, using infrared spectroscopy, between the carbon nanotubes and inorganic covering materials [16]. The iron oxide particles adhere to the surface of oxidized MWCNTs and also form small agglomerations as free particles. Overall, the iron oxide nanoparticles seem to be distributed uniformly on the surface of the MWCNT.

**Figure 4:**
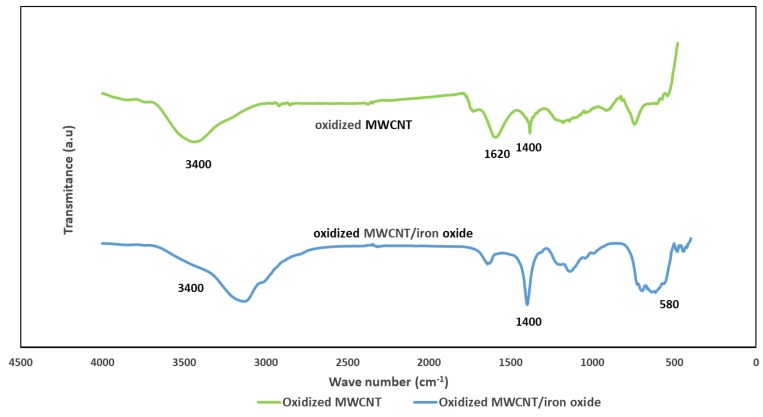
FTIR spectrum of the oxidized MWCNT and the oxidized MWNT/iron oxide composite.

The bonding status of the oxidized MWCNT and MWCNT/iron oxide composite was checked using the wavelength-dependent transmittance data obtained using an FTIR spectrometer (Thermo Scientific, Waltham, MA, USA). Figure 4 shows the FTIR spectra for the oxidized MWCNT and oxidized MWCNT/iron oxide composite, respectively. The bands at around 1620 cm^−1^ in the spectra of both materials are associated with the C=C vibrations, which result from the inherent structure of the nanotubes [15]. The absorption peak corresponding to the stretching vibration of OH (~3400 cm^−1^) indicates that oxygenated groups were produced on the surface of MWCNT after the acid treatment. The peaks at 1400 cm^−1^ are attributed to the C-OH stretching and O-H bending vibrations. Compared to the oxidized MWCNT, this peak increased in the spectrum of oxidized-MWCNT/iron oxide. As reported by other researchers, C=O vibration of the carboxyl (COOH) group was observed at 1735 cm^−1^ for oxidized MWCNT, and it disappeared from the MWCNT/iron oxide composite spectra. This could be the result of interactions between the negative charge from the carboxyl groups and positive charge from the iron oxide nanoparticles [17,18]. In the spectra of the oxidized MWCNT/iron oxide, the clear broadband around 580 cm^−1^ is due to the interaction of Fe-O-Fe and shows the presence of gamma iron oxide [17,18,19].

**Figure 5:**
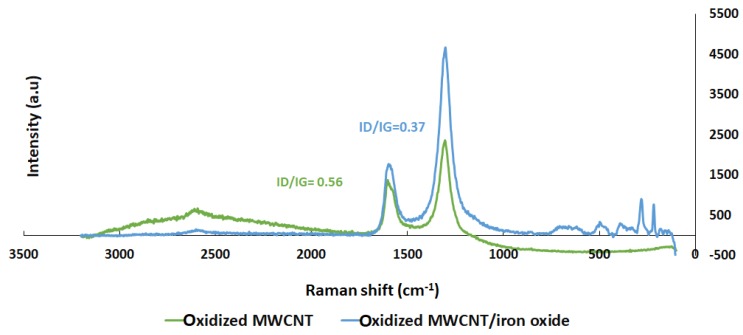
Raman spectra of oxidized MWCNTs and the oxidized MWNT/iron oxide composite.

Figure 5 shows the Raman spectra of both the oxidized MWCNT and the MWCNT/iron oxide composite. The Raman spectra were recorded at room temperature by a Renishaw Raman spectrometer with a linear laser excitation of 785 nm (He-Ne). For each sample, exposed for 10 s, three distinct points were measured and displacement occurred between 100 and 1400 cm^−1^. It is well known that the sharp band at 1590 cm^−1^ (G band) is attributed to the in-plane vibration of the C–C bond, while the band at 1350 cm^−1^ (D band) is attributed to activation by the presence of disorder in the carbon systems [19,20]. The second-order peak at ~2750 cm^−1^ is called 2D band. The Raman spectrum of oxidized MWNT/iron oxide showed a strong band at 671 cm^−1^, 489 cm^−^1 and 280 cm^−1^, which are characteristic peaks of iron oxide nanoparticles [20,21]. It was also observed that the ratio of the the intensities of D band and G band (ID/IG) of oxidized MWCNT has been changed from 0.56 to 0.37 with the addition of iron oxide. The decrease in this ratio indicates that the atomic ordering of the MWCNT was enhanced and defects were reduced. This suggests that the iron oxide nanoparticles formed chemical bonds with the oxidized MWCNT surface [22].

### 3.3. Sensing Results

**Figure 6:**
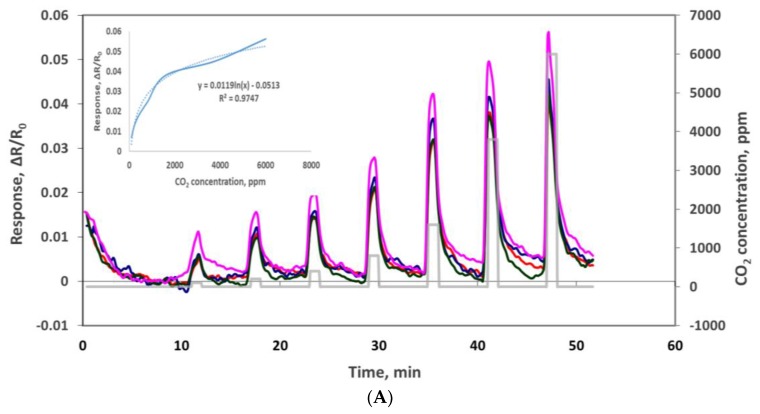

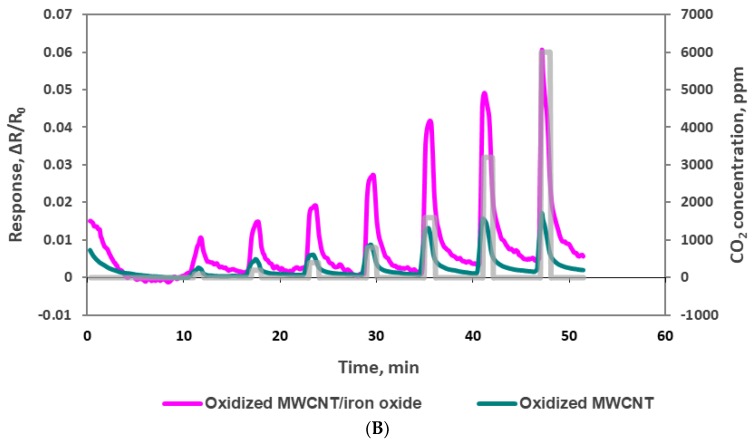
(**A**) Responses (ΔR/R_o_) to 100, 200, 400, 800, 1600, 3800 and 6000 ppm CO_2_ with oxidized MWCNT/iron oxide nanocomposite sensors. (**B**) Comparison of oxidized MWCNT/iron oxide nanocomposite and oxidized MWCNT response to 100, 200, 400, 800, 1600, 3800 and 6000 ppm CO_2_.

The electrical resistance of the sensors at baseline and as sensor responses to CO_2_ were measured using the experimental set-up depicted in Figure 1. Oxidized MWCNT-based sensors have average base resistance around 1300 Ω, and the oxidized MWCNTs/iron oxide (3.5% by weight) composite-based sensors have an average base resistance around 13,000 Ω. The sensor chip was exposed, at room temperature, to CO_2_ concentrations of 100, 200, 400, 800, 1600, 3800 and 6000 ppm at the interval stated in the Experimental section and as shown in the Figure 6. The response is normalized resistance (*R*–*R*_0_/*R*_0_), where *R*_0_ is the sensor base resistance with no CO_2_ exposure and R is the resistance at time t with CO_2_ exposure. We can see that the sensor resistance increased when it was exposed to various concentrations of CO_2_ gas, and this change was concentration dependent. Sensor response to the same sensing material channels is very similar (see Figure 6A) and the slight variations might be due to the manual deposition process. Figure 6B shows the response curves from sensors made by two different sensing materials. From the response curves of the oxidized MWCNT sensor channels and the oxidized MWCNT/iron oxide channels, we observed that the response of the latter was improved greatly (≈5 times). This may be due to the availability of the two possible locations for CO_2_ molecules adsorption, either at the MWCNT surface or at the iron oxide nanoparticles, which is a different sensing mechanism with the composite material [23,24]. As reported previously, hybrid metal oxide decorated carbon nanotubes showed an enhanced response as a gas sensing material due to a mechanism that induces a modulation of surface charges. Metal oxide has mainly n-type semiconductor characteristics and MWCNT have p-type semiconductor characteristics. These differences result in two depletion regions formed in such hybrid films. The first depletion region is located at the metal oxide surface and the second one is located at the interface between the metal oxide nanoparticle and the MWCNT. The adsorption of the CO_2_ molecule induces a modulation of surface charges that directly influences the electrons transfer between the heterojunctions and induces a variation in the resistance of the sensing layer [25]. Also, the presence of MWCNT in the iron oxide matrix can introduce nanochannels. These nanochannels play an important role in the gas diffusion process. The gas molecules can easily transport into the gas-sensing layers via these nanochannels, leading to increased sensitivity [26,27]. Similar results of enhanced responses have been reported with metal oxide and carbon nanotubes-based composite materials [23,24,25,26,27,28,29]. In addition, with the addition of iron oxide, the sensor response range seems to be increased. The oxidized MWNT-based sensor was saturated at 1600 ppm CO_2_, while the composite material-based sensor showed concentration dependence up to 6000 ppm CO_2_.

**Figure 7:**
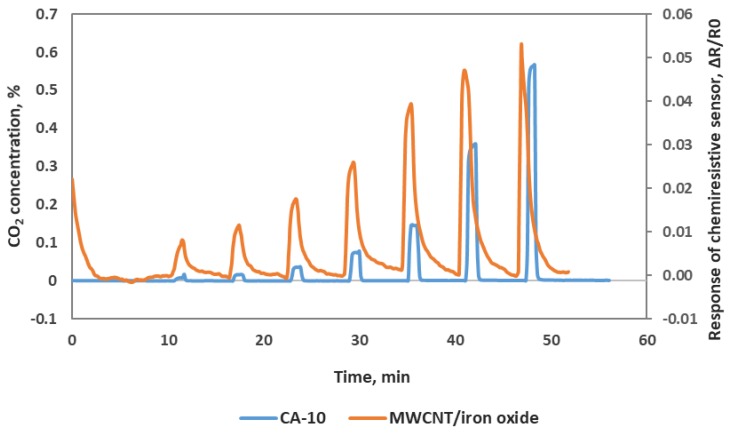
Oxidized MWCNT/iron oxide chemiresistive sensor response along with CA-10 CO_2_ analyzer response.

We also confirmed our chemiresistve sensor reponse with a commercial benchtop instrument—Sable System’s CA-10 analyzer. This comparison assured the input CO_2_ concentrations of the sensor testing and also confirmed our sensor performance. We used a T-joint at the outlet of the Environics gas mixing system and allowed one stream to flow to the chemirestive sensor and another stream to CA-10 CO_2_ analyzer. We can see in Figure 7 that the chemiresistive sensor response and recovery cycles match well with the CA-10 analyzer response pattern to CO_2_. In Figure 7, the blue line of the response curve was obtained from the CA-10 CO_2_ analyzer in which the peaks corresponding to the CO_2_ concentrations are shown on the left Y-axis, and the orange line of the response curve was obtained from our MWCNT/iron oxide sensor with a relative resistance change (right Y-axis) to the exposure of different CO_2_ concentrations. Our chemiresistive sensor takes about 10 s to reach a plateau that is comparable with the CA-10 CO_2_ analyzer. The accuracy of the concentration measurements of our sensor is also comparable with the commercial instrument. The response of our chemiresistive sensor to lower CO_2_ concentrations is more sensitive compared to the commercial CO_2_ analyzer.

**Figure 8:**
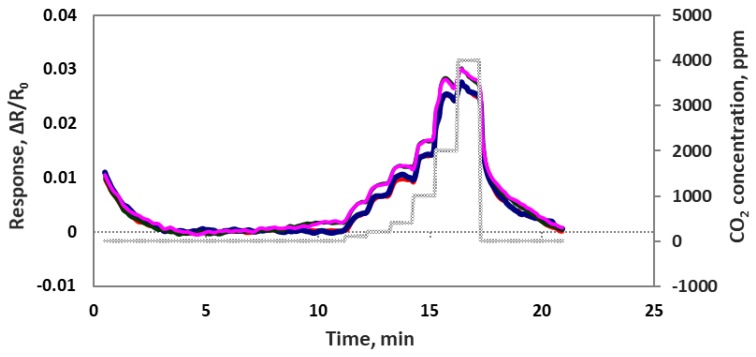
Four individual oxidized MWCNT/iron oxide composite sensor responses (ΔR/R_o_) to the step input of 100, 200, 400, 1000, 2000 and 4000 ppm CO_2_.

**Figure 9:**
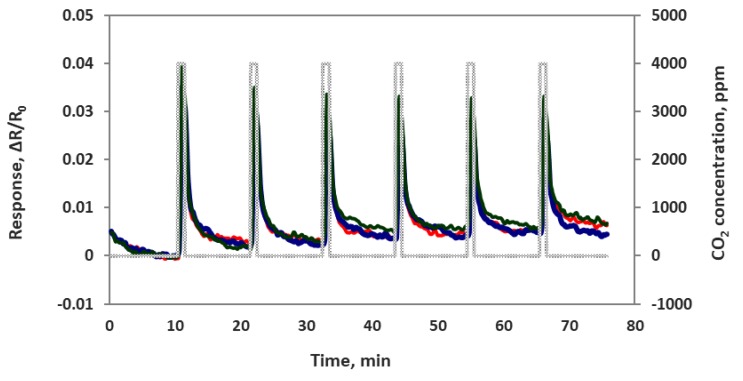
Composite material-based sensor responses (ΔR/R_o_) to muiltiple exposure of 4000 ppm CO_2_.

We also checked sensor performance for the case of increasing the CO_2_ concentration continuously step by step without air purge in between, as seen in Figure 8. Sensors were exposed to 100, 200, 400, 1000, 2000 and 4000 ppm concentrations each for 1 min at room temperature. The sensor response time was very fast at about 5–10 s. All 4 sensor channels showed very similar responses to each other, which means these sensors are reproducible. In another experiment we checked the sensor’s repeatability by exposing the sensor chip multiple times to 4000 ppm of CO_2_. As we can see in Figure 9, the sensor chip showed a good response to 4000 ppm CO_2_, but the sensor response to the first CO_2_ exposure was higher than the rest of the CO_2_ exposures. This is normal chemiresitive sensor behavior and it shows that the sensor needs a warm up time to achieve a stable performance.

### 3.4. CO_2_ Sensor Selectivity and Humidity Dependence

**Figure 10:**
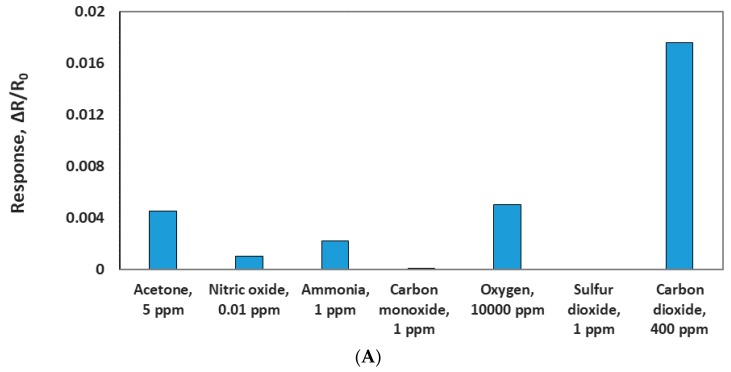

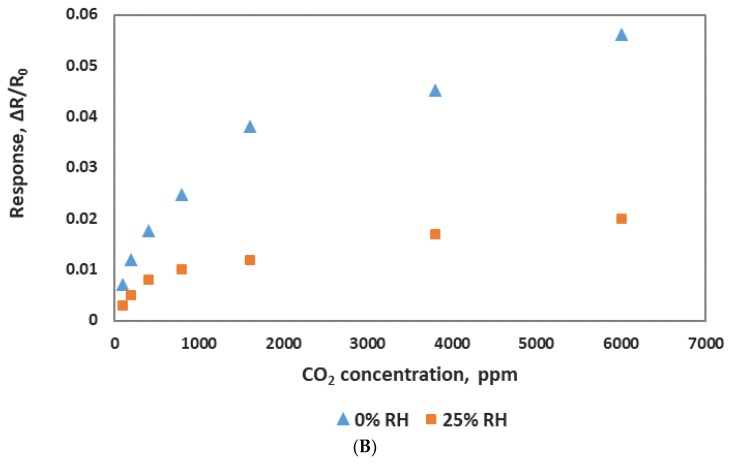
(**A**) Comparison of the sensor responses of CO_2_ and other gases. (**B**) Sensor response to various concentrations of CO_2_ at two different relative humidities (RH).

To evaluate the selectivity of the MWCNT/iron oxide, the sensor chip was tested against 5 ppm acetone, 0.01 ppm nitric oxide, 1 ppm ammonia, 1 ppm carbon monoxide, 10,000 ppm oxygen, and 1 ppm sulfur dioxide in dry air at room temperature. These concetrations were chosen based on the most common concetrations of these species found in ambient air. Sensor response to 400 ppm CO_2_ (presented in ambient air) was used to compare its response to these possible interferences, as seen in Figure 10A. The sensor showed some responses to few gases. However, these responses are negligible compared to the 400 ppm CO_2_ response. This indicates negligible cross sensitivity and adequate selectivity.

Humidity’s effect on the sensor response was investgated at room temerature (25 °C) with various levels of relative humidity (RH). The sensor chip was exposed to different concentrations of CO_2_ at a fixed value of humidity. As shown in Figure 10B, sensing response varied with the humidity and was significantly reduced at 25% RH. Sensors showed almost no response at 50% RH. This could be the result of water molecules blocking the CO_2_ adsoption on the surface of the sensing material. The dependence of sensor response on humidity seems to be a problem, but it can be addressed by using hydrophobic nanotubes or other techniques such as membrane filtration, dessicant drying and freezing seperation to remove the humidity effect.

### 3.5. CO_2_ Sensor Intergration with A Smartphone

**Figure 11:**
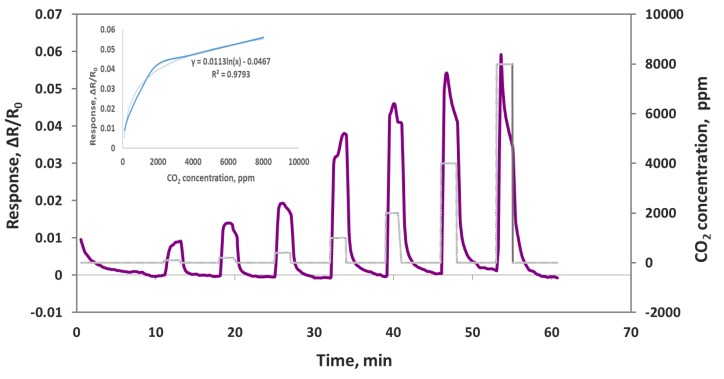
Oxidized MWNT/iron oxide composite sensor responses (ΔR/R_o_) to 100, 200, 400, 1000, 2000, 4000 and 8000 ppm CO_2_ on a smartphone device.

As we reported in our previous publications [30,31], we have developed a sensor module that can be integrated with a smartphone, and it can sniff out trace amounts of gases in real time. Smartphone sensors have many advantages, such as low cost, compactness, low power consumption, easy operation, and network sensing capability. We deposited the composite sensing material of oxidized MWCNT/iron oxide onto a smartphone sensor chip that can be plugged into the sensor module. This sensor module was than plugged into a smartphone with an application that we developed for sensor data acquisition, storage and processing. The smartphone sensor was exposed to CO_2_ concentrations in the range of 100–8000 ppm, and the sensor responses were obtained, as shown in the Figure 11. The CO_2_ gas exposure was 2 min each, and the air purge was 10 min in the beginning and 5 min between CO_2_ exposures. The smartphone sensor showed a very good response to CO_2_ with quick response and recovery time in seconds. The corresponding calibration curves are shown in the inset. Using the same sensing material of MWCNT/iron oxide, a sensor chip measured to detect CO_2_ with a Keithley instrument and with the smartphone platform confirms that our chemiresistive sensor indeed works for CO_2_ detection.

## 4. Conclusions

In this study, a chemiresistive sensor comprising of oxidized MWCNT and nanoparticles of iron oxide has been prepared and used as a sensing material for CO_2_ detection at room temperature. It was found that adding a small amount of iron oxide nanoparticles to MWCNT, we can enhance the sensitivity to CO_2_ by ~5 times, expand the detection range of the CO_2_ concentration from 100 ppm to 6000 ppm, have quick response and recovery in 10 s, and have good repeatability from measurement to measurement and good reproducibility from sensor to sensor. As reported by many researchers, this enhanced sensitivity is a result of nano-heterojunction formation at the interface between nanotubes and iron oxide nanoparticles. The presence of nano-heterojunctions induces a modulation of surface charges. In addition to the presence of carbon, nano-channels in the metal oxide enhance gas adsorption. These two mechanisms increase the sensor sensitivity manifold. We also calibrated the sensor chip with a commercial CO_2_ analyzer and our chemiresistive sensor showed comparable detection capability with advantages of smaller size, lower power, low cost and the ability to be easily multiplexed and integrated with existing electronics. We have also demonstrated CO_2_ detection using a smartphone sensing device that can be used for wireless and network sensing.

Our future work will focus on developing a methodology to pre-treat the sensor in order to shorten the warm up time. Based on the results of the humidity study, we need to remove the moisture from CO_2_ in the air stream.
